# Accurate Quantitative Histomorphometric-Mathematical Image Analysis Methodology of Rodent Testicular Tissue and Its Possible Future Research Perspectives in Andrology and Reproductive Medicine

**DOI:** 10.3390/life12020189

**Published:** 2022-01-27

**Authors:** Réka Eszter Sziva, Júlia Ács, Anna-Mária Tőkés, Ágnes Korsós-Novák, György L. Nádasy, Nándor Ács, Péter Gábor Horváth, Anett Szabó, Haoran Ke, Eszter Mária Horváth, Zsolt Kopa, Szabolcs Várbíró

**Affiliations:** 1 Department of Obstetrics and Gynecology, Semmelweis University, Üllői Street 78/a, 1082 Budapest, Hungary; acsjulia97@gmail.com (J.Á.); acs.nandor@med.semmelweis-univ.hu (N.Á.); varbiro.szabolcs@med.semmelweis-univ.hu (S.V.); 2 Department of Physiology, Semmelweis University, Tűzoltó Street 37-47, 1094 Budapest, Hungary; nadasy.gyorgy@med.semmelweis-univ.hu (G.L.N.); simoncorbel@126.com (H.K.); horvath.eszter@med.semmelweis-univ.hu (E.M.H.); 3 Workgroup of Research Management, Doctoral School, Semmelweis University, Üllői Street 22, 1085 Budapest, Hungary; 4 2nd Department of Pathology, Semmelweis University, Üllői Street 93, 1091 Budapest, Hungary; tokesa1972@yahoo.co.uk; 5 Department of Pathology, Géza Hetényi Hospital, Tószegi Street 21, 5000 Szolnok, Hungary; novak.agnes@gmail.com; 6 Budapest 3rd District General Practitioner, Hegyestű Bt., Füst Milán Street 28, 1039 Budapest, Hungary; dr.horvath.peter.gabor@gmail.com; 7 Andrology Centre, Department of Urology, Semmelweis University, Üllői Street 78/b, 1082 Budapest, Hungary; a.szabo1995@gmail.com (A.S.); kopa.zsolt@med.semmelweis-univ.hu (Z.K.)

**Keywords:** quantitative histomorphometry, mathematical image analysis, method, digital pathology, testis, research, andrology, reproductive medicine, future

## Abstract

Infertility is increasing worldwide; male factors can be identified in nearly half of all infertile couples. Histopathologic evaluation of testicular tissue can provide valuable information about infertility; however, several different evaluation methods and semi-quantitative score systems exist. Our goal was to describe a new, accurate and easy-to-use quantitative computer-based histomorphometric-mathematical image analysis methodology for the analysis of testicular tissue. On digitized, original hematoxylin-eosin (HE)-stained slides (scanned by slide-scanner), quantitatively describable characteristics such as area, perimeter and diameter of testis cross-sections and of individual tubules were measured with the help of continuous magnification. Immunohistochemically (IHC)-stained slides were digitized with a microscope-coupled camera, and IHC-staining intensity measurements on digitized images were also taken. Suggested methods are presented with mathematical equations, step-by-step detailed characterization and representative images are given. Our novel quantitative histomorphometric-mathematical image analysis method can improve the reproducibility, objectivity, quality and comparability of andrological-reproductive medicine research by recognizing even the mild impairments of the testicular structure expressed numerically, which might not be detected with the present semi-quantitative score systems. The technique is apt to be subjected to further automation with machine learning and artificial intelligence and can be named ‘Computer-Assisted or -Aided Testis Histology’ (CATHI).

## 1. Introduction

Infertility incidence and prevalence are increasing worldwide, up to 15% of reproductive-aged couples are affected by infertility according to the World Health Organization [1].

According to the International Glossary on Infertility and Fertility Care, infertility can be defined as: ‘a disease characterized by the failure to establish a clinical pregnancy after 12 months of regular, unprotected sexual intercourse or due to an impairment of a person’s capacity to reproduce either as an individual or with his/her partner. Fertility interventions may be initiated in less than 1 year based on medical, sexual and reproductive history; age; physical findings and diagnostic testing. Infertility is a disease, which generates disability as an impairment of function.’ [2]. 

In Europe, the cause of infertility is unknown in 10–15% of infertile couples, and among those cases, male factors can be identified in approximately 45–50% [3]. Thus, basic, translational and clinical research in the field of andrology and reproductive medicine are of the utmost importance. Histopathological evaluation of testicular tissue in these studies is also of great importance and can provide valuable information about spermatogenesis and fertility. Beside these research studies, in case of diagnostic andrological evaluation, human testicular histological sampling can improve our knowledge of male infertility.

Nowadays, from a clinical diagnostic point of view, several 1 to 10 or 0 to 10 points score systems have been developed based on the microscopic view of the testicular tissue, such as the Johnsen score [4], Modified Johnsen or de Kretser and Holstein score [5] and the Bergmann-Kliesch score [6,7]. The Johnsen score evaluates all of the tubular section in one section of the sample and gives scores from 1–10 according to the number and presence or absence of spermatozoa, spermatids, spermatocytes, spermatogonia, Sertoli cells and organization of the germinal epithelium [4], while the modified Johnsen or De Kretser and Holstein score introduced the concept of complete, normal and incomplete spermatogenesis and differentiated between late and early spermatids [7]. The Bergmann-Kliesch score compares the number of tubules containing elongated late spermatids and sperm to the total number of tubules on the sample and gives 0–10 points according to the calculated percentages [6,7]. These score systems are semi-quantitative and do not provide exact quantitative numerical details of the structure of the spermatogenic epithelium, tubules or testis tissue.

However, quantitative histometric or morphometric analysis of the testicular tissue is not an unknown field of andrology. In as early as the 1950s–1990s, numerous light and electron microscopic methods were developed, mostly points- or lines-signed microscope eyepieces with fix magnification and a predetermined number of investigated live cross-sectioned microscopic fields were used to identify or score the different components of animal and human testicular tissue in various andrological conditions. The distinct measured or counted components were seminiferous tubules; germinal or spermatogenic epithelium and lumen; spermatogenic cell types (spermatogonia, spermatocytes, spermatids); different cycles, stages and kinetics of the seminiferous epithelium; Sertoli cells; interstitial and connective tissue; intertubular space cell types (e.g., Leydig cells, fibroblasts) and other distinct cells and debris [8,9,10,11,12,13,14,15,16,17,18,19,20,21,22,23,24,25,26,27,28,29,30,31,32,33,34,35,36,37,38,39,40].

These microscopic methods were complicated, heterogenous and time-consuming, although really creative and innovative at the time. 

Later, the improvement of microscope and computer techniques made it possible to create a microscope-coupled camera, and several image analysis programs have been developed which allowed the examination of the live microscopic images. Furthermore, capture of these live microscopic images on fixed magnifications (5×, 10×, 20×, 40×, 100× or higher) resulted in digitized images where several various quantitative measurements on testicular components could be taken for further research purposes [41,42,43,44,45,46]. However, in order to investigate the whole testicular tissue, a huge number of digitized images would be necessary. Moreover, these evaluation techniques are not uniform; thus, the results are not completely comparable.

Here we would like to present and describe a new, useful and accurate quantitative histomorphometric-mathematical image analysis methodology of testicular tissue, which could be used not only in basic animal and translational research but which could hopefully, in the future, help the human clinical diagnostics and may contribute to the future research perspectives of andrology and reproductive medicine. As basic and translational andrological researches are often carried out on rodents nowadays, we used rodent testicular tissue from one of our previous experiments; however, our technique could also be easily applied to human testicular tissue. 

## 2. Materials and Methods: Basic Methodology and Conceptions

### 2.1. Rodent Testicular Histology Sections for Quantitative Image Analysis 

#### 2.1.1. Animals, Standard and Immunohistochemical Stainings

Different vitamin D status was induced in 22 4-week-old male Wistar rats in an 8-week-long experiment. The control group (*n* = 11) received conventional vitamin D containing rat chow with additional vitamin D supplementation, whereas vitamin D deficient (VDD) animals (*n* = 11) received a special vitamin D-free diet [47]. After 8 weeks, serum 25-hydroxivitamin D levels indicated a significant, fivefold reduction in the VDD group compared with the control animals [48]; these results validated our model. 

The testes of the sacrificed animals were removed. After weighing them, the testes were freshly fixed with 4% paraformaldehyde, embedded in paraffin. Native and standard hematoxylin-eosin (HE) stained sections were made. HE-stained sections from different groups were used to present our accurate quantitative histomorphometric-mathematical image analysis measurement, and native slides were stained against VDR and MAGE-A4 to present our immunohistochemical evaluation method. 

#### 2.1.2. Digitization of the Stained Testis Tissue Cross Sections

**Figure 1:**
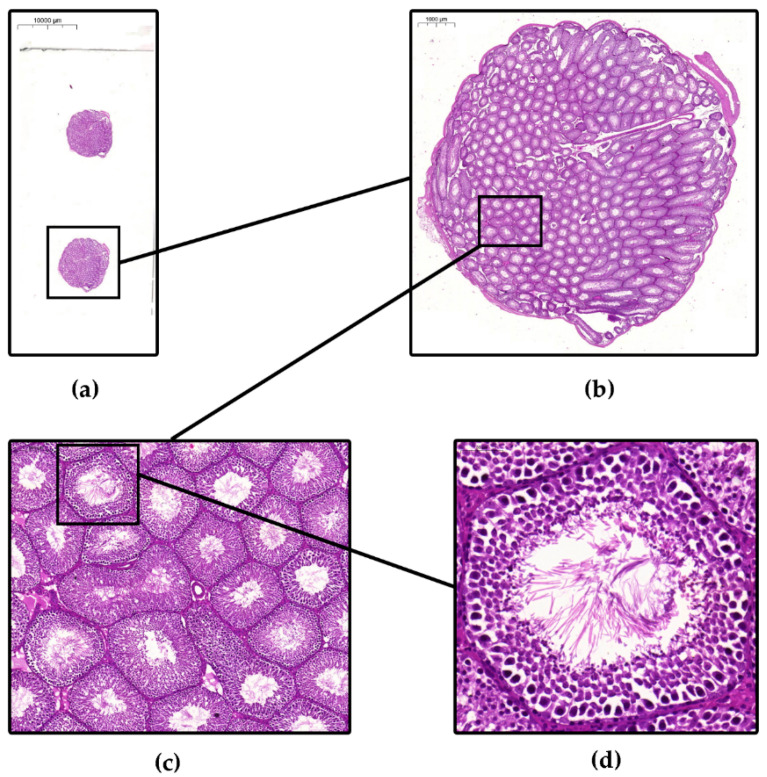
Representative images of Hematoxylin-Eosin-stained testis tissue cross sections from the control group at different magnifications. (**a**) Whole scanned slide with two testis tissue cross sections, scale bar: 10,000 μm; (**b**) A whole testis tissue cross section, scale bar: 1000 μm; (**c**) Magnified cross- and longitudinal-sectioned seminiferous tubules, scale bar: 200 μm and (**d**) Single cross-sectioned seminiferous tubule with sperm cells in the lumen, scale bar: 50 μm. Images were captured with the ‘Take a snap’ tool of the CaseViewer program.

HE-stained cross sections were digitized and scanned with slide-scanner (PANNORAMIC^®^ 1000 DX, 3DHISTECH Ltd., Budapest, Hungary) and a free-access slide-viewing software from the same firm, CaseViewer, was used (CaseViewer 2.3.0.99276, 3DHISTECH Ltd., Budapest, Hungary) in order to allow visualization and evaluation of the whole testis cross section (Figure 1). 

**Figure 2:**
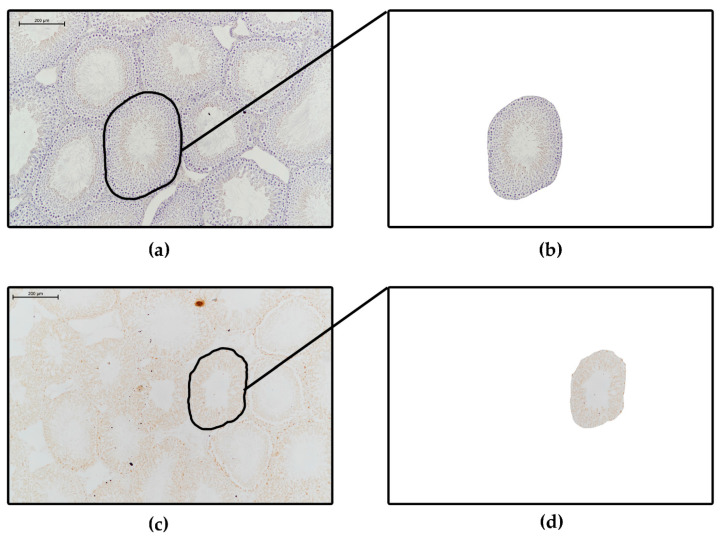
Representative images of immunohistochemically stained testicular tissue. (**a**,**b**) Stained against vitamin D receptor protein with Hematoxylin-counterstaining (Control group). (**c**,**d**) Stained against MAGE-A4, a specific spermatogonium marker protein (Vitamin D deficient group). The images were taken with a Nikon Eclipse Ni-U microscope coupled with a Nikon DS-Ri2 camera using NIS Elements BR and Image J 1.50b image analysis software. The brown color shows DAB chromogen-positivity which indicates receptor-positivity, and the blue/violet color represents hematoxylin counterstaining. (**b**,**d**) Single tubules from (**a**,**c**) panels without background, the previously round-selected tubules have been cleared of background with the ‘Image/Clear outside’ tool from the Image J program. 10× objective, scale bars: 200 μm.

Immunohistochemical (IHC) VDR- and MAGE-A4-stained sections were digitized with a microscope-coupled camera (Nikon Eclipse Ni-U, 933584 microscope with Nikon DS-Ri2 camera, NIS Elements BR image software, Nikon Corporation, Minato City, Tokyo, Japan). Randomized pictures were taken on 10× magnification and each picture represented at least 6–8 whole seminiferous tubules. Fourteen pictures were made of each testis per group. The use of microscope-coupled camera-made images is beneficial for the immunohistochemical staining intensity measurements because the microscope-coupled camera provided high-resolution images of the staining of the tubules. This method allowed the visualization of at least 80–100 pieces of IHC-stained whole tubules in the case of rat testis (Figure 2). 

### 2.2. Quantitative Histomorphometric-Mathematical Image Analysis Measurements on Digitized Pictures

For our novel quantitative histomorphometric-mathematical image analysis measurements the slide-viewing CaseViewer software was used (CaseViewer 2.3.0.99276, 3DHISTECH Ltd., Budapest, Hungary). This program allows the use of any magnification (Figure 1a–d) and can measure the concrete area, perimeter, length and ‘thickness’, in square-micrometers (μm^2^) and micrometers (μm), of any organ or tissue compartment on the scanned slides with the appropriate settings and calibration. This method ensures that the whole testis cross section of typical laboratory animals, which are commonly used in andrological basic and translational research (e.g., rodents, rats, mice) or even human testis tissues from biopsies or surgical-microsurgical operational procedures, can also be visualized, continuously magnified and quantitatively measured and evaluated. Another benefit of the program is that the measured data are exportable into an Excel-file for further computations.

#### 2.2.1. Geometrical Measurements on Scanned Standard Hematoxylin-Eosin-Stained Slides

The visibility of the whole testis tissue or biopsy cross sections provides the possibility to count and measure several accurate histomorphometric-mathematical parameters. 

The testis can be divided into *capsule*, *seminiferous tubules* and *interstitium*. A single seminiferous tubule can be divided further into *lamina basalis* and *seminiferous* or *spermatogenic epithelium (SE)* with *intratubular lumina* [33]. The cross section of the seminiferous tubule can be further subdivided into lamina basalis, Sertoli cells, Sertoli cell nucleus, spermatogonia, spermatocytes, round spermatids, elongated spermatids and tubular lumen [49]. The interstitium contains peritubular myoid cells, blood vessels, collagen fibers, intercellular space, Leydig cells, macrophages and lymphatic vessels [33]. However, the condition of spermatogenic epithelium of one tubule is affected by the particular stage of spermatogenic wave, the characteristics of which are species-specific [50,51].

In accordance with the morphometric model, the following histomorphometric-mathematical parameters can be determined: 

##### Total Seminiferous Tubule Number

**Figure 3:**
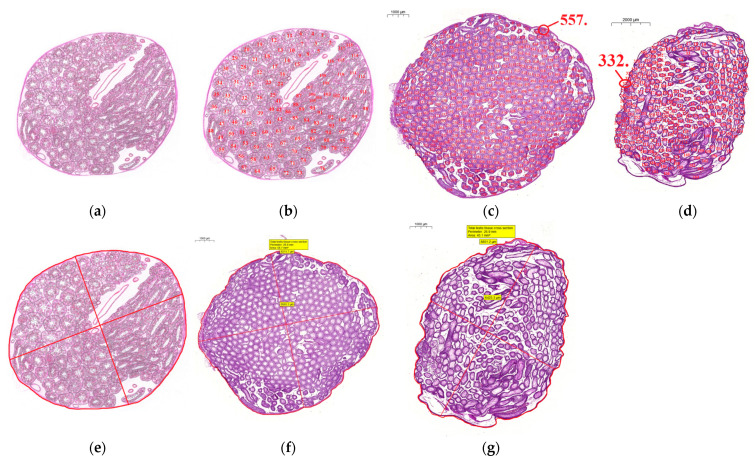
Total seminiferous tubule-counting and measuring the geometrical parameters of the testis: total testis tissue cross-section area, perimeter and average total testis tissue diameter measurements. (**a**,**b**,**e**) Schematic simplified drawings of laboratory animal testis cross sections illustrating tubule-counting and area, perimeter and diameter measurement. (**c**,**d**) Realization of total seminiferous tubule number determination: in our (**c**) Control group, scale bar: 1000 μm, n_(all counted tubules)_ = 557; and in our (**d**) VDD group, scale bar: 2000 μm, n_(all counted tubules)_ = 332. (**f**,**g**) Realization of total testis tissue cross-section area, perimeter and average total testis tissue diameter measurements: in the (**f**) Control group, scale bar: 1000 μm, and in the (**g**) VDD group, scale bar: 1000 μm. Measured data can be read in yellow brackets. The drawings are hand-made, whereas in case of the images, CaseViewer and Microsoft Paint programs were used.

We can count all seminiferous tubules (both cross- and longitudinal-sectioned tubules) on the cross section of the testis tissue. One can use the ’Take a snap’ option of the program or take a ‘PrintScreen’ on a certain magnification where the whole testis cross section and all of the tubules can be seen well and use Microsoft Paint software (Microsoft Corporation, Redmond, WA, USA) to make JPEG or TIFF images. Then each tubular cross section can be numbered (unit: pieces). It can serve also as an internal control (Figure 3b–d).

##### The Counting of ‘Elongated Spermatid-Positive’ Seminiferous Tubules to Determine ‘Histomorphometric’ Bergmann-Kliesch Percentage Score

Having counted all of the seminiferous tubules, we can determine which tubules contain elongated spermatids (spermatid cells which have an elongated head and are no longer attached to spermatogenic epithelium) indicating spermatogenesis and which do not; thus ‘Histomorphometric’ Bergmann-Kliesch score can be calculated:

(1) $$‘\text{Histomorphometric}’\;\text{Bergmann}-\text{Kliesch}\;\text{score}\;\text{percentage}\;(\%)\;=\frac{‘\text{Elongated}\;\text{spermatid}-\text{positive}’\;\text{seminiferous}\;\text{tubules}\;(\text{pieces})}{\text{Total}\;\text{seminiferous}\;\text{tubule}\;\text{number}\;(\text{pieces})}\times 100.\;$$

Internal control images are also useful to sign the ‘elongated spermatid-positive’ tubules.

##### Total Testis Tissue Cross-Section Area, Perimeter and Average Total Testis Tissue Diameter

With accurate manual round-selection of the whole testis tissue cross section, CaseViewer program measures area (unit: μm^2^) and perimeter (unit: μm).

The two longest perpendicular (e.g., horizontal and vertical) diameters (unit: μm) can also be measured, and using the average calculation, the average total testis tissue diameter (unit: μm) can be defined (Figure 3e–g):

(2) $$\text{Average}\;\text{total}\;\text{testis}\;\text{tissue}\;\text{diameter}\;(\mu m)=\;=\frac{\text{Total}\;\text{testis}\;\text{tissue}\;\text{diameter}\;{\left(\mu m\right)}_{1}+\text{Total}\;\text{testis}\;\text{tissue}\;\text{diameter}\;{\left(\mu m\right)}_{2⊥1}}{2}$$

##### Random Choice of Predetermined Number of Seminiferous Tubule Cross-Sections for Further Detailed Investigation

To get an even deeper insight into the morphology of individual tubules, we have to determine how many tubules should be included in the study and choose a random selection of them. Longitudinally-sectioned tubules are inappropriate for these measurements. In case of animal studies, the number depends on the size of the animals (e.g., rat testes are larger than mouse testes). We can perform preliminary power-analysis to determine the number of tubules to be tested or just choose 75–100 pieces of seminiferous tubules per experimental animal to ensure adequate sampling.

One further complication is that human testis biopsy and histological samples from surgical-microsurgical operations are much smaller than a whole rodent testis cross section. In this case it is advantageous to include all available cross-sectioned tubules in the sample.


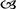
 Area and perimeter measurements, calculations

**Figure 4:**
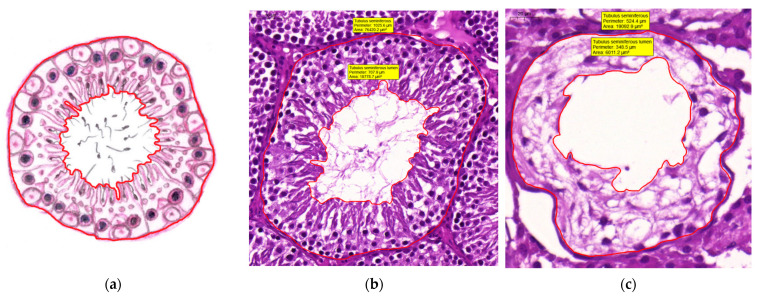
Geometry of seminiferous tubules: area and perimeter. (**a**) A schematic drawing showing how to outline the contour. (**b**,**c**) Realization: Outer and inner contours of seminiferous tubules and their lumen, measuring tubule and lumen area (**b**) in the Control group, scale bar: 50 μm and (**c**) in the VDD group, scale bar: 20 μm. Measured data can be read in yellow brackets. From the area data, spermatogenic epithelium area can be calculated. The CaseViewer and Microsoft Paint programs were used.

With accurate manual round-selection of the ‘cell-border’ of a tubule and the lumen of the tubule, we can determine area and perimeter characteristics of one single tubule (Figure 4a–c):

- Seminiferous tubule area (μm^2^) and perimeter (μm)
- Seminiferous tubule lumen area (μm^2^) and perimeter (μm)
  In case of tubular area measurement, we have to be careful to identify the tubule we have chosen and do not include the tubule wall. Otherwise repeated measurements can deform the statistics. In addition, when assigning the lumen of a tubule, we should precisely follow the luminal border. Although clear limits can be determined in certain cases, it is also possible that the borders of the lumen cannot be distinguished clearly from the spermatogenic epithelium (e.g., head of the sperm is still in Sertoli cell—in the spermatogenic epithelium—but the tail belongs to the lumen already). In this case, it might help if we consider the border to be the neck or midpiece of the sperm which is already separated from Sertoli cells.
- Spermatogenic epithelium area (μm^2^)
  From the abovementioned measured characteristics of a tubule, we can calculate the spermatogenic epithelium area of one particular tubule if we extract the lumen-area from the tubule-area of the tubule in question by using the following equation:
  (3) $$\text{Spermatogenic}\;\text{epithelium}\;\text{area}\;({\mu m}^{2})=\;=\text{Seminiferous}\;\text{tubule}\;\text{area}\;{\left({\mu m}^{2}\right)}_{\text{identical}}\;-\text{Seminiferous}\;\text{tubule}\;\text{lumen}\;\text{area}\;{\left({\mu m}^{2}\right)}_{\text{identical}}$$
- Spermatogenic epithelium area ratio (%)
  A ratio of spermatogenic epithelium area and tubule-area of the same seminiferous tubule gives us the area percentage of the spermatogenic epithelium:
  (4) $$\text{Spermatogenic}\;\text{epithelium}\;\text{area}\;\text{ratio}\;(\%)\;=\frac{\text{Spermatogenic}\;\text{epithelium}\;\text{area}\;{\left({\mu m}^{2}\right)}_{\text{identical}}}{\text{Seminiferous}\;\text{tubule}\;\text{area}\;{\left({\mu m}^{2}\right)}_{\text{identical}}}\;\times \;100$$

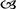
 ‘Distance’ measurements: Diameter and ‘Thickness’

**Figure 5:**
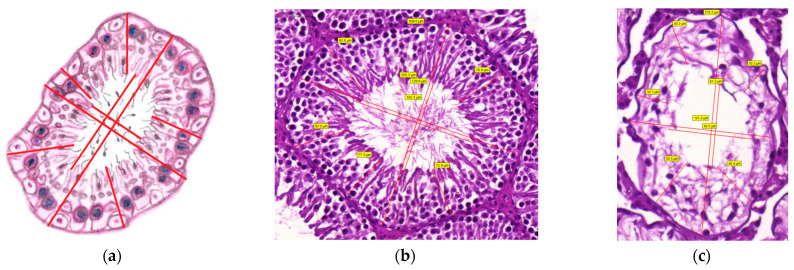
Quantitative evaluation of seminiferous tubule geometry: diameter and spermatogenic epithelium thickness. (**a**) A schematic drawing showing how to measure these parameters. (**b**,**c**) Realization: Outer and inner perpendicular diameters of seminiferous tubules and spermatogenic epithelium thicknesses: (**b**) in the control group, scale bar: 50 μm and (**c**) in the VDD group, scale bar: 20 μm. Measured diameter and thickness data are shown in yellow brackets. The CaseViewer and Microsoft Paint programs were used.

By measuring the two perpendicular diameters (unit: μm) of the tubule and its lumen, we can calculate (Figure 5a–c):

- Average seminiferous tubule diameter (μm)
  (5) $$\text{Average}\;\text{seminiferous}\;\text{tubule}\;\text{diameter}\;(\mu m)=\;=\frac{\text{Seminiferous}\;\text{tubule}\;\text{diameter}\;{\left(\mu m\right)}_{1}+\text{Seminiferous}\;\text{tubule}\;\text{diameter}\;{\left(\mu m\right)}_{2⊥1}}{2}$$
- Average seminiferous tubule lumen diameter (μm)
  (6) $$\text{Average}\;\text{seminiferous}\;\text{tubule}\;\text{lumen}\;\text{diameter}\;(\mu m)=\;=\frac{\text{Seminiferous}\;\text{tubule}\;\text{lumen}\;\text{diameter}\;{\left(\mu m\right)}_{1}+\text{Seminiferous}\;\text{tubule}\;\text{lumen}\;\text{diameter}\;{\left(\mu m\right)}_{2⊥1}}{2}$$

The ‘thickness’ of the spermatogenic epithelium (SE) is also a measurable parameter. For accurate sampling, choose five different points of spermatogenic epithelium randomly where we can measure length (in μm):

- Average spermatogenic epithelium thickness (μm)
  (7) $$\text{Spermatogenic}\;\text{epithelium}\;(\mathrm{SE})\;\text{thickness}\;(\mu m)=\;\frac{\mathrm{SE}\;\text{length}\;{\left(\mu m\right)}_{1}+\;\mathrm{SE}\;\text{length}\;{\left(\mu m\right)}_{2}+\;\mathrm{SE}\;\text{length}\;{\left(\mu m\right)}_{3}+\;\mathrm{SE}\;\text{length}\;{\left(\mu m\right)}_{4}+\;\mathrm{SE}\;\text{length}\;{\left(\mu m\right)}_{5}}{5}\;$$

##### Other Useful Measurements

- Total interstitial and other tissue area (μm^2^)
  We can also discover some useful and interesting pieces of information about the possible changes of the interstitial components of testis tissue if we measure the area of all of the seminiferous tubules (in this case, the cross and longitudinal-sectioned tubules also) and extract it from total testis tissue cross-section (CS) area:
  (8) $$\text{Total}\;\text{interstitial}\;\text{and}\;\text{other}\;\text{tissue}\;\text{area}\;{\left({\mu m}^{2}\right)}_{\mathrm{CS}}=\;=\;\text{Total}\;\text{testis}\;\text{tissue}\;\text{area}\;{\left({\mu m}^{2}\right)}_{\mathrm{CS}}\;-\text{Seminiferous}\;\text{tubule}\;\text{area}\;{\left({\mu m}^{2}\right)}_{\text{All}\;\text{countable}\;\text{tubules}\;\text{on}\;\mathrm{CS}}$$
- Total seminiferous tubule area ratio (%)
  We can correlate the area of all of the seminiferous tubules (cross and longitudinal-sectioned tubules) with the total testis tissue cross-section area and with this calculation we receive information about what percentage of the testicular tissue area is occupied by tubular area:
  (9) $$\text{Total}\;\text{seminiferous}\;\text{tubule}\;\text{area}\;\text{ratio}\;{\left(\%\right)}_{\mathrm{CS}\;}\;=\frac{\text{Seminiferous}\;\text{tubule}\;\text{area}\;{\left({\mu m}^{2}\right)}_{\text{All}\;\text{countable}\;\text{tubules}\;\text{on}\;\mathrm{CS}}}{\text{Total}\;\text{testis}\;\text{tissue}\;\text{area}\;{\left({\mu m}^{2}\right)}_{\mathrm{CS}}}\times 100$$
- Total seminiferous tubule number ratio (pieces/μm^2^)
  With the following method, we can normalize the counted tubules data to the size of the investigated testis tissue cross section, so the different-sized testis tissue cross sections can be comparable:
  (10) $$\text{Total}\;\text{seminiferous}\;\text{tubule}\;\text{number}\;\text{ratio}\;(\text{pieces}/{\mu m}^{2}{)}_{\mathrm{CS}}=\;=\frac{\text{Total}\;\text{seminiferous}\;\text{tubule}\;\text{number}\;{\left(\text{pieces}\right)}_{\text{All}\;\text{countable}\;\text{tubules}\;\text{on}\;\mathrm{CS}}}{\text{Total}\;\text{testis}\;\text{tissue}\;\text{area}\;{\left({\mu m}^{2}\right)}_{\mathrm{CS}}}$$

#### 2.2.2. Staining Intensity and Density Measurements on Digitalized Immunohistochemically-Stained Slides

**Figure 6:**
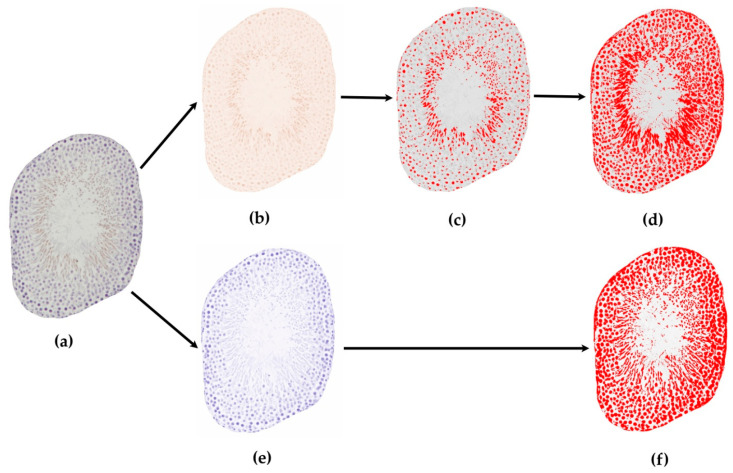
Immunohistochemistry for the VDR protein with DAB (brown) color development, counterstained with hematoxylin (blue) in an isolated seminiferous tubule cross section image from the Control group and the measurement of Vitamin-D-receptor (VDR)-positive pixel area. (**a**) Image of the VDR immunohistochemistry of an isolated tubule counterstained with hematoxylin (identical with Figure 2b, Control group). (**b**) Separation of DAB brown and (**e**) Hematoxylin blue colors. (**c**,**d**,**f**) 8-bit grey-scale images of tubule from the adequate stained part (same row, (**b**–**d**): DAB, (**e**,**f**): Hematoxylin): (**c**) Threshold where the VDR-positively stained pixels are red, (**d**) Threshold where all DAB-stained pixels are red—for mean pixel value measurement for non-calibrated optical density (**f**) Threshold where all Hematoxylin-stained pixels are red (counterstaining).

**Figure 7:**
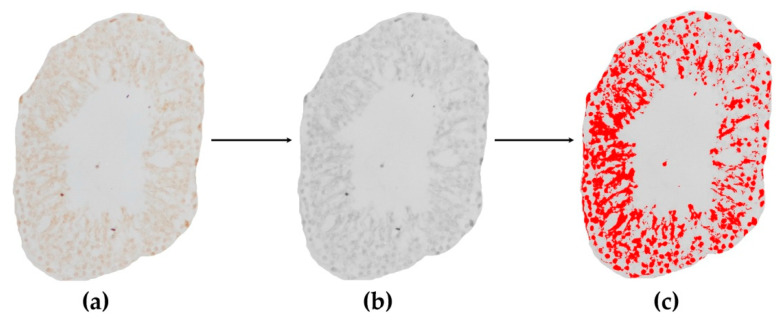
Immunohistochemistry for MAGE-A4 with DAB (brown) color development without counterstaining in an isolated seminiferous tubule cross section image from the VDD group and measurement of MAGE-A4-positive mean pixel value. (**a**) Original DAB-colored tubule (identical with Figure 2d), (**b**) its 8-bit grey-scale transformation and (**c**) threshold where all DAB-positively stained pixels are red—for mean pixel value measurement for non-calibrated optical density.

With immunohistochemical staining of any tissue, receptor expression and other levels of expressed materials can be evaluated. To quantitatively determine the level of expression of the tested receptors or other materials we can use another free software, Image J or the newer version, FIJI (ImageJ 1.50b or 1.52i, National Institutes of Health, Bethesda, MD, USA). During the immunohistochemical staining, the use of chromogens—such as brown-colored diamino-benzidine (DAB) or black-colored nickel-DAB (Ni-DAB)—is essential, while counterstaining is optional (usually Hematoxylin in case of DAB or Nuclear Fast Red in case of Ni-DAB). Here, we present both staining types: testis tissue with and without counterstaining (VDR and MAGE A4 staining, Figure 6 and Figure 7).

When using counterstaining, with ‘Colour deconvolution’ plugin, the program disassembles our original DAB- and Hematoxylin-stained immunohistochemical image by color. Generally, DAB staining is brown-colored and the color of Hematoxylin is blue or violet (if counterstaining was used, and the third image is complementary of the first two colors, usually light green or nothing/white). For immunostained slides, the ‘H DAB’ or ‘FastRed/FastBlue/DAB’ option may be adequate, but the possibility of disassembling many other types of staining is also available in the program. Specific basic testis cell-markers, such as MAGE-A4 for spermatogonia, WT-1 and SF-1 for Sertoli cells and INSL-3 for Leydig cells allow us to examine or count concrete cell types. It is also important to use such specific immunohistochemical markers or their combinations, which indicate the early/round, late/elongated spermatids and sperms (e.g., DOG1), to help to find possibly usable cells in different histopathological conditions such as maturation arrest, tubular fibrosis, mixed atrophy and Sertoli-cell-only syndrome [5,52].

- Randomly choosing a predetermined number of evaluable seminiferous tubules or other parts of the testicular tissue.
  For accurate evaluation of the level of expression, we randomly have to choose a predetermined number of both cross-sectioned and longitudinal-sectioned tubules from previously digitized pictures. In case of tubules, choosing only cross sections may simplify the evaluation. In the program we can manually round-select each evaluable tubule and cut it out from the original picture by clearing the background (Figure 2a,b). This can be done for each predetermined number of evaluable tubules. Thus, with this method we have only one single tubule in one picture without any background to distract from the tubule, and now we can disassemble the single stained tubule by color with the ‘Colour deconvolution’ plugin. Logically, a brown-colored picture shows DAB-staining that indicates the positively-stained area or components of the tubule, and a blue- or violet-colored picture represents the counterstained background (Figure 6a,b,e). By converting the colored pictures into 8-bit gray-scale images (‘Image/Type/8 bit’ option), we can set a threshold (‘Image/Adjust/Threshold’ option) for the stained structures in each picture, which has been selected by the program, usually with a red color (Figure 6b–f).
  With regard to determining the threshold (0–255) for the positively-stained area (DAB picture), it is advantageous to compare the threshold-selected positively-stained area on the DAB 8-bit gray-scale image with the original un-disassembled picture (only positively-stained components should be red) (Figure 6c compared to Figure 6a).
  To outline the total stained area, we should convert the counterstained blue picture into 8-bit grey-scale image and use the threshold-setting to select the entire stained area (all components are red) (Figure 6a,e,f). After threshold-setting, using the ‘Analyze/Measure’ function offered by the program, the stained area can be measured (in pixel) on each image and from the measured data, the positively-stained area percentage can be calculated if we compare the positively-stained area to the total stained area (Figure 6c compared to Figure 6f):
- Positively-stained area percentage (%):
  (11) $$\mathrm{Positively}\text{-}\text{stained}\;\text{area}\;\text{percentage}\;(\%)\;=\frac{\mathrm{Positively}\text{-}\text{stained}\;\text{area}\;{\left(\text{pixel}\right)}_{\text{area}\;\text{in}\;\mathrm{DAB}\;\text{brown}\;8\text{-}\text{bit}\;\text{image}}}{\text{Total}\;\text{stained}\;\text{area}\;{\left(\text{pixel}\right)}_{\text{area}\;\text{in}\;‘H’\;\text{or}\;‘\text{FastBlue}’\;8\text{-}\text{bit}\;\text{image}}}\times 100$$

Additionally, the program also measures mean pixel values on the 8-bit images. From the mean pixel value, which was measured on DAB 8-bit image by thres- hold setting (Figure 6d), uncalibrated optical density of the positively-stained structures can be calculated with an equation recommended by Image J:

- Uncalibrated optical density/OD (Arbitrary Unit):
  (12) $$\text{Uncalibrated}\;\text{optical}\;\text{density}\;(A.U.)=\log_{10}(\frac{255}{{\text{Mean}\;\text{pixel}\;\text{value}}_{\mathrm{DAB}\;\text{brown}\;8\text{-}\text{bit}\;\text{image}}})$$

## 3. Application of Our Technique: Realization and Implementation

Before any histological examination, careful measurements of the removed animal testis (or of human biopsy material) should be performed: sample size in 3 dimensions, color and any abnormal visible difference or impairment on its surface should be photographically documented and the wet and dry weight of the samples should be measured—as in a routine clinical pathological examination. In case of animal experiments, we have the possibility to cut and embed samples from different parts of the same testis (e.g., proximal, median and distal); the contralateral testis can be embedded in a longitudinal direction to examine single tubules lengthwise. 

In the following representative images, we demonstrate our carefully applied quantitative histomorphometric-mathematical image analysis method of the testis cross-sectional tissue. (At the beginning of our method-development, we measured most of the described quantitative parameters on our test tissue: testis cross sections of Vitamin D supplemented and Vitamin D deficient rats, *n* = 8/groups. Our graphical and numerical data can be found in the ‘Supplementary Materials’ paragraph. According to our negative results, we hypothesize that; short-term (8-week-long) Vitamin D deficiency does not affect the quantitative-mathematical parameters or the structure of the spermatogenic testicular tissue.)

### 3.1. Digitization of Hematoxylin-Eosin- and Immunohistochemically-Stained Slides

In Figure 1, the continuous magnification of a scanned slide of HE-stained rat testicular tissue cross-section can be seen. Each picture represents a different magnification stage and both the overview of the cross-section (structure of the tubules and the vascular and connective tissue) and the details of a single tubule (arrangement of the spermatogenic epithelium, containing the tubule lumen and single sperm and other spermatogenic line cells) can also be seen (Figure 1a–d).

In Figure 2, VDR and MAGE-A4 immunohistochemically-stained rat testicular tissue can be seen on 10× magnifications from our different groups. In the first column (Figure 2a,c), on the original images, we indicated the detailed manual round-selection of the previously picked single tubules in each picture; thus, in the second column, the previously round-selected single tubules appear totally cleared from the background, so the positively-stained area and staining-intensity measurements can be done on a single tubule.

### 3.2. Quantitative Image Analysis of Histological Sections

#### 3.2.1. Geometrical Measurements on Scanned Standard Hematoxylin-Eosin-Stained Slides

In Figure 3, first, we present a schematic drawing of a testis cross-section (Figure 3a) to allow the visualization of geometrical measurements (Figure 3b: total seminiferous tubule counting, Figure 3e: total testis tissue cross-section area, perimeter and average total testis tissue diameter measurements), then the described real measurements can be seen on the cross-sections derived from our experimental animals (Figure 3c,d,f,g).

Figure 4 and Figure 5 show the geometrical measurements of a single tubule; first the methods are visualized on a schematic drawing (Figure 4a and Figure 5a), after which the execution of the measurements on tubules of experimental rats can be seen. (Figure 4b,c and Figure 5b,c).

#### 3.2.2. Quantitative Measurements on Digitalized Immunohistochemically-Stained Slides

Figure 6 shows an immunohistochemically-stained single tubule (from Figure 2b) stained against Vitamin D receptor, visualized with DAB and counterstained with Hematoxylin (Figure 6a). Color separation was achieved with the help of an image-analyzer program (Figure 6b: DAB, Figure 6e: Hematoxylin). The grey-scale image of the brown DAB channel was used to measure VDR-DAB-positive pixels for positively-stained area measurement (Figure 6c) and for all DAB-positive pixels for optical density measurement (Figure 6d), and finally, the grey-scale image of the hematoxylin background was used for positively-stained area measurement (Figure 6f). 

Figure 7 indicates MAGE-A4 immunohistochemical staining of a single tubule without counterstaining (from Figure 2d and Figure 7a), and its grey-scale version, which was used to determine all DAB-positive pixels for optical density measurement (Figure 7b,c).

## 4. Discussion and Conclusions

As far as we know, this is the first paper that provides accurate methodological instructions for the quantitative and mathematical image analysis measurements of testis tissue and makes use of some of the benefits of digital technology (scanning, continuous magnification, easy handling of histological sections, image and staining intensity analysis). Furthermore, it presents the suggested methods with detailed description of the utilized mathematical equations and illustrates them with representative images. The detailed, step-by-step description of our method makes it possible for other research teams to use the described evaluations. Our methodology forms a new research technique; at the moment it yields new possibilities for different scientific tasks in the field of andrology. Additional histological-immunohistochemical evaluations, such as quantitative identification of histologically- or immunohistochemically-labeled spermatogenic or other cell types (e.g., spermatogonia, Sertoli cells, Leydig cells) and other tissue components (e.g., connective tissue elements, myoid cells, blood or lymphatic vessels), can be planned and performed in an analog manner. 

In the early years of the development of histomorphometrical testis tissue analysis, several creative solutions were found: usage of points- or lines-signed microscope eyepieces with fix magnification and of a predetermined number of investigated live cross-sectioned microscopic fields. With these options, different components of the testicular tissue can be identified or scored. First, Roosen-Runge EC et al. applied the quantitative volumetric analysis method of Chalkley HW on human testicular biopsies of normal and different infertility-categorized (infertility with azoospermia, with oligo- or normospermia, before and after nitrofuran treatment, infertility with no sperm count) testes and determined the relative volume of the following testicular components: interstitial tissue, basement membrane and tunica propria; Leydig cells; spermatogonia; spermatocytes; spermatids and spermatozoa; abnormal germ cells; Sertoli cells; lumen; ‘space’ and total germ cells [8,9]. Chalkley HW described a method in 1943, which made the volumetric analysis of the tissue possible by five pointers-signed 10× eyepiece: four points were the ‘recorders’ which made ‘hits’ and the fifth one was the ‘focuser’. Investigation of 5 × 35 microscopic fields with 91× oil-immersion objective was determined to be sufficient for statistical purposes [8]. Later, Schöffling K et al. applied another histometric examination [13] to study the tubules and interstitial tissues of diabetic rats, diabetic Chinese hamsters and ob/ob-mice [10,11]. In this case, researchers used an integrated 15- or 25- point-marked microscope eyepiece and Hennig’s point-counting histometric method from 1958 [10,13,53] for quantitative analysis. The ocular points were arranged in a circle to cover the examined area and the tissue component under each point was counted as a ‘hit’ on 20× magnification in a previously determined number of selected microscopic fields (15–20). Germinal epithelium, lumen and interstitial tissue [10] or in another division, tubules, interstitial cells and connective tissue [13,24] have been distinguished, and for each tissue component, the number of hits was summarized and correlated to the total number of the counted hits or to the hits of different component making ratios. Dykes JRW has already applied this quantitative histomorphometric method for human testicular biopsies of normal and several andrological malconditions (chromatin-negative and -positive Klinefelter Syndrome, maldescended, true undescended-type, atrophic testes, cases of maturation arrest and mild primary gonadal deficiency) and approved its accuracy, reproducibility and significance in the case of differentiation in distinct pathological impairments of the testes [13]. Mancini E et al. also described another quantitative method; they investigated testicular biopsies in different age groups of unilateral cryptorchidism-cases and used various histological staining for distinction between different cell and tissue component types. From circular transverse-sectioned seminiferous tubules, 50 were selected for counting and the average number of different cell types (germinal epithelium and Sertoli cell lines, intertubular space cell types—fibroblasts, Leydig cells and degenerating cells and stomal connective tissue) per 50 tubules was determined and the diameter of the tubules was measured also with exclusion of the basement membrane [12]. Meanwhile, Clermont Y et al. determined the cycle and stages of the seminiferous epithelium in different laboratory animals [54,55,56,57,58] and in humans [15,17], which further described the kinetics of the spermatogenesis in mammals [16]. However, in the end, these difficult, time-consuming microscopic points- or lines-signed eyepieces methodologies were not used widely. Moreover, the lack of archiving of the evaluated images further deteriorated the reproducibility of these methods. 

The invention of the microscope-coupled camera and its combination with computer image-analyzer programs offered opportunities of the investigation of the real, fix-magnified, microscopic images of testicular tissue and semen smears. Sukura A et al. investigated the total tissue area; seminiferous tubule and interstitium area; Leydig and Sertoli cell numbers and different cell-density in spermatogenic epithelium in a particular field. And further morphological parameters (area, lengths, axes) of spermatozoa have been additionally determined, but these measurements performed only on six, randomly collected tissue-section images of both-side testicles from each animals [41]. Herrera-Alarcon J et al., in four quadrants of fix-magnified digital testicular images, evaluated the volume percentage and tubular components of the testicular parenchyma and counted the tubules with and without lumen and the elongated spermatid-containing tubules. With the help of an analysis software they calculated several variables (area, perimeter, minor-major diameters) from the number of measured tubules. They investigated four randomly selected areas representing each quadrant of lamb’s testicular cross-section [42]. Ma L et al. counted and calculated the volume fractions (proportions) of different testicular structures (volume of the seminiferous tubule, interstitial tissue, tubule lumen, length and diameter of tubules, thickness of seminiferous epithelium) with (9 or 20) computer-generated point-hitting technique on systematically-sampled fields of digital testicular sections and their total volumes were further estimated [43]. Zamani A et al., with the point-counting method on fix-magnified digitized pictures, estimated the volume densities, and the total volumes of seminiferous tubules, interstitial tissue, and germinal epithelium, numerical densities of Leydig, Sertoli and germ cells were also calculated [59]. But point-hitting techniques are a little bit antique in the time of the microscopic image digitization and measurements [43]. Nihi F et al. used camera-coupled high-resolution light microscopy and transmission electron-microscopy with image-analysis software to identify the germ cells and determine the duration of the stages of the human seminiferous epithelium cycle [44]. Xu J et al. developed a computerized spermatogenesis staging system to determine and subclassify the stages of the spermatogenic cycle [60]. Umar Z et al. used Image J on fix-magnified photomicrographs of testis tissue to determine the diameter, area, luminal diameter and spermatogenic cell layer of seminiferous tubules and the thickness of the germinal layer [45]. Kazemi S et al. used another software to measure, on fix-magnified images, the perpendicular diameters, surface area of tubules and epithelium height, to determine the number of spermatogonia and spermatocytes and the number of seminiferous tubules in 1 cm^2^ [46]. According to previously well-described digital histomorphometrical measurement methods reported by former researchers, we planned and implemented a complex and detailed methodology: we determined multiple measurable and calculable parameters not only on the tubular level but on the whole testis cross-sectional level at the same time. Dumont L et al., based on the microscope-coupled image-making method, has already developed an automated digital immunohistochemistry image analysis tool to reduce the background and noise, to generate stitched high-resolution images of testis sections and to extract the necrotic area and to count the immunostained cells [61]. 

Digital pathology and histology have been undergoing huge developments recently, several benefits can be claimed: scanned and digitized slides can be accessed regardless of place, time and equipment (computer, phone, tablet instead of microscope); they have portability, shareability, easy-handling, available at any time, standardization and usability in any field of medicine [62,63]. The utilization of artificial intelligence and deep machine-learning coupled with microscopic techniques in reproductive medicine and assisted reproductive techniques (oocyte, sperm and embryo assessment) is increasingly popular [64,65]. Nowadays, 2-dimensional serial-sectioning in combination with computer software, 3-dimensional reconstruction of several organs and tissues, such as the testicular cord or seminiferous tubules became possible [66]. The combination of digital histology-pathology, artificial intelligence and machine-learning can provide promising diagnostic and therapeutic opportunities in various medical specialties. 

Benefits of slide-scanning technique is not unknown in andrology, similar ones have been used for quantitative sperm morphology analysis [67,68] which could be complemented with the help of convolutional neuronal network deep-learning [69]. A supervised machine learning-based prediction model that has been developed for the identification of patients with Klinefelter-syndrome among azoospermic patients, has significantly better sensitivity and can improve diagnostic rate of the illness [70]. Another deep learning-based method was developed to assess the stages of Wistar rat spermatogenic cycle on hematoxylin-eosin-stained digital slides, which makes the quick evaluation of stage-frequency possible [71]. Deep learning can be used in the classification of the immunohistochemistry images of human testis and improve the diagnostic performance [72]. 

Although our quantitative histomorphometric-mathematical image analysis is still a manual evaluation, the accurate counting and geometrical measurements can serve as a basis for later automation. The computer-based identification of testicular tissue structures and cell types with the application of artificial intelligence and deep machine learning techniques under strict human supervision can be implemented in analogy with the abovementioned examples and Computer-Assisted or -Aided Sperm Analysis (CASA) [73]. Various automated semen quality analysis systems exist and demonstrate highly concordant results, which can be compared to manual analyzing methods [74].

From the clinical point of view, our method may provide an approach for the differential diagnosis of certain histopathological conditions of infertility (hypospermatogenesis, different levels of maturation arrest, tubular fibrosis, mixed atrophy and Sertoli-cell-only syndrome). For instance, in the case of maturation arrest, the elongated/late spermatids are absent, correspondingly the number/ratio of ‘elongated spermatid-positive’ seminiferous tubules or ‘Histometric’ Bergmann-Kliesch percentage score would be lower. In tubular fibrosis, the tubules are constricted and disappeared; therefore, the number, area, perimeter and diameter of the tubules would reduce while the interstitial tissue amount would relatively increase as the result of fibrosis. In Sertoli-cell-only syndrome, the spermatogenic epithelium is severely reduced or absent, leading to a greater area, perimeter, diameter of tubular lumen and a smaller thickness of the spermatogenic epithelium. 

In conclusion, our novel quantitative histomorphometric-mathematical image analysis method can improve the reproducibility, objectivity, quality and comparability of basic, translational and clinical histologic-histomorphometric research and investigations of testicular tissues. It may contribute to the recognition of mild, initial impairments and differences of testicular structure, providing precise values of measurable parameters, increasing the sensitivity for the distinction of animal experimental and human study groups which may not be achieved with semi-quantitative score systems. Additionally, with the accumulation of experience, a clinical-diagnostic application can be developed in the near future that can recognize and quantitatively characterize pathologic situations with much higher accuracy compared to the semi-quantitative systems presently used. Furthermore, additional immunohistochemical staining can be used to identify the presence of possibly usable spermatids and sperms for assisted reproductive techniques. In the future, our evaluation, automated with the help of artificial intelligence and deep machine learning, could be named ‘Computer-Assisted or -Aided Testis Histology (CATHI)’.

Limitations of our study: we demonstrated the suggested methods on rat testicular tissue instead of human testicular biopsy samples; thus, further basic, translational and clinical human studies are required to confirm our method.

## Data Availability

The data which are additionally presented in this methodological article are openly available in our [Supplementary Materials].

## Supplementary Materials

The following supporting information can be downloaded at: https://www.mdpi.com/article/10.3390/life12020189/s1, Figure S1-Table S1: Total seminiferous tubule number; Figure S2-Table S2: Average seminiferous tubule area; Figure S3-Table S3: Average seminiferous tubule lumen area; Figure S4-Table S4: Average seminiferous tubule perimeter; Figure S5-Table S5: Average seminiferous tubule lumen perimeter; Figure S6-Table S6: Average calculated spermatogenic epithelium area; Figure S7-Table S7: Average calculated spermatogenic epithelium area ratio; Figure S8-Table S8: Average seminiferous tubule diameter; Figure S9-Table S9: Average seminiferous tubule lumen diameter; Figure S10-Table S10: Average spermatogenic epithelium thickness; Picture S1-S2: Representative histological picture of testicular tissue from our groups. Figure S11-Table S11: Vitamin D Receptor (VDR)-positively-stained area percentage.
