# Supplementary material for: Accurate Quantitative Histomorphometric-Mathematical Image Analysis Methodology of Rodent Testicular Tissue and Its Possible Future Research Perspectives in Andrology and Reproductive Medicine
Source: Life (Basel). 2022 Jan 27;12(2):189. doi: 10.3390/life12020189 (PMC8875546; doi:10.3390/life12020189)
Supplement: Supplementary file 1 [file life-12-00189-s001.zip › life-1415659-supplementary.pdf]

Supplementary Materials

In addition to

'Accurate Quantitative Histomorphometric-Mathematical Image Analysis Methodology of Rodent Testicular Tissue and its Possible Future Research Perspectives in Andrology and Reproductive Medicine'

Original article

Statistical Analysis

Measurements were carried out between November 2019 and October 2020. Some details of the methodology has to be presented on the first-corresponding author's (Réka Eszter Sziva MD PhD) PhD Complex Examination on 10 June 2020 during her PhD studies at the PhD School of Semmelweis University, Károly Rácz Clinical Medicine Doctoral School, Budapest, Hungary.

Statistical analysis and figures were made with the help of GraphPad Prism 7.0 (GraphPad Software, San Diego, CA, USA) software. After checking Kolmogorov-Smirnov, D'Agostino and Pearson omnibus and Shapiro-Wilk normality tests, in case of data with normal distribution, we used parametric unpaired T-test with F-test, data with non-normal distribution were analyzed with non-parametric Mann–Whitney U-test. $p < 0.05$ was considered statistically significant for all statistical analyses. Values with normal distribution are expressed as Mean ± SEM, data with non-normal distribution are expressed as Median [IQR].

• ***Total seminiferous tubule number*** (pieces)

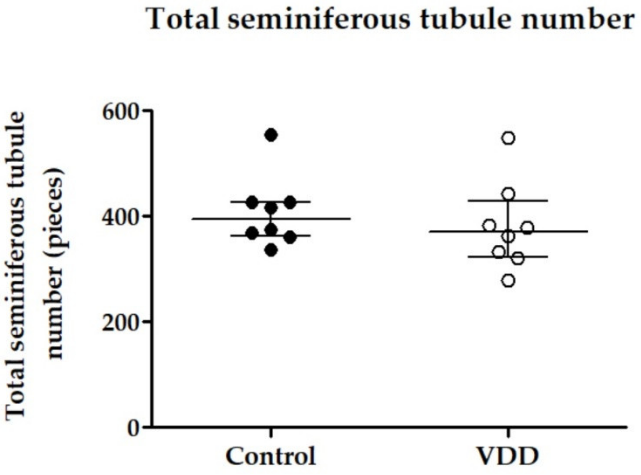

Control/Vitamin D supplemented group (n = 8, pieces)	Vitamin D deficient/VDD group (n = 8, pieces)
426	443
416	279
361	383
426	332
554	321
374	549
369	378
337	363

Figure S1 and Table S1: ***Total seminiferous tubule number*** per group: Figure S1: in graph, Table S1: original data. Abbreviations: VDD = vitamin D deficient. n = 8/group. Mann-Whitney-U-test, data are in Median [IQR], n.s.

• *Average seminiferous tubule and seminiferous tubule lumen area* (µm²)

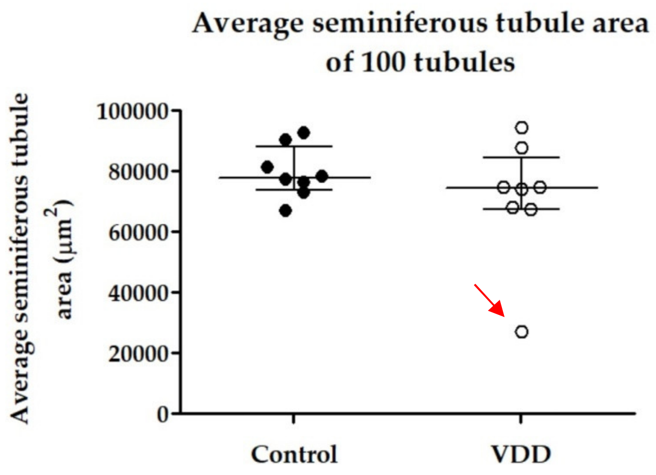

Control/Vitamin D supplemented group (n = 8, µm²)	Vitamin D deficient/VDD group (n = 8, µm²)
77392.840	87900.100
90568.170	74809.390
73110.510	74950.830
78453.180	68048.630
92981.960	94346.640
76410.900	27258.600
81535.660	67501.790
67238.510	74183.800

Figure S2 and Table S2: *Average seminiferous tubule area* per group: Figure S2: in graph, Table S2: original data. Abbreviations: VDD = vitamin D deficient. n = 8/group. Mann-Whitney-U-test, data are in Median [IQR], n.s.

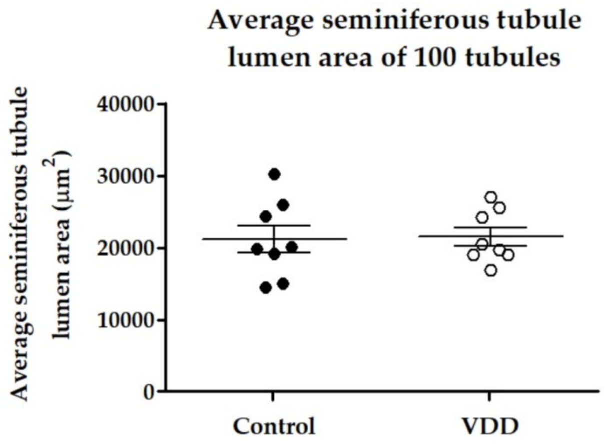

Control/Vitamin D supplemented group (n = 8, µm²)	Vitamin D deficient/VDD group (n = 8, µm²)
15044.930	24255.690
30195.800	20463.890
20081.520	19062.920
19773.420	26990.820
24390.210	25501.990
19143.340	16833.180
25901.760	19020.470
14508.560	19669.120

Figure S3 and Table S3: *Average seminiferous tubule lumen area* per group: Figure S3: in graph, Table S3: original data. Abbreviations: VDD = vitamin D deficient. n = 8/group. Unpaired-t-test, data are in Mean±SEM, n.s.

• *Average seminiferous tubule and seminiferous tubule lumen perimeter* (µm)

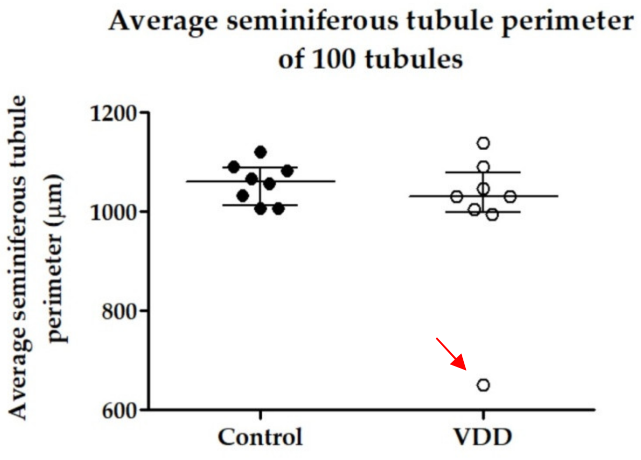

Control/Vitamin D supplemented group (n =8, µm)	Vitamin D deficient/VDD group (n = 8, µm)
1006.332	1090.215
1121.538	1047.698
1056.021	1030.083
1066.135	1005.809
1082.678	1138.890
1032.218	650.730
1091.270	995.647
1006.779	1030.562

Figure S4 and Table S4: *Average seminiferous tubule perimeter* per group: Figure S4: in graph, Table S4: original data. Abbreviations: VDD = vitamin D deficient. n = 8/group. Mann-Whitney-U-test, data are in Median [IQR], n.s.

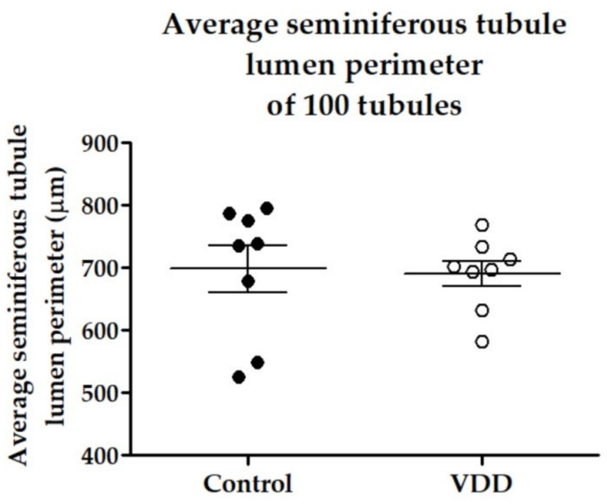

Control/Vitamin D supplemented group (n = 8, µm)	Vitamin D deficient/VDD group (n = 8, µm)
525.708	714.927
787.231	694.579
738.916	632.222
735.737	733.656
795.629	768.397
679.924	581.973
775.971	698.079
549.257	702.360

Figure S5 and Table S5: *Average seminiferous tubule lumen perimeter* per group: Figure S5: in graph, Table S5: original data. Abbreviations: VDD = vitamin D deficient. n = 8/group. Unpaired-t-test, data are in Mean±SEM, n.s.

• *Average calculated spermatogenic epithelium area and area ratio* (µm² and %)

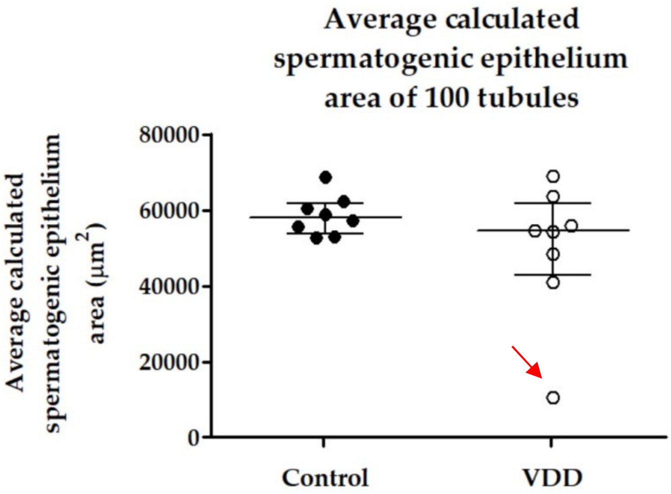

Control/Vitamin D supplemented group (n = 8, µm²)	Vitamin D deficient/VDD group (n = 8, µm²)
62347.910	63644.410
60372.370	54345.500
53028.980	55887.910
58679.770	41057.800
68591.760	68844.650
57267.560	10425.420
55633.900	48481.320
52729.940	54514.680

Figure S6 and Table S6: ***Average calculated spermatogenic epithelium area*** per group: Figure S6: in graph, Table S6: original data. Abbreviations: VDD = vitamin D deficient. n = 8/group. Mann-Whitney-U-test, data are in Median [IQR], n.s.

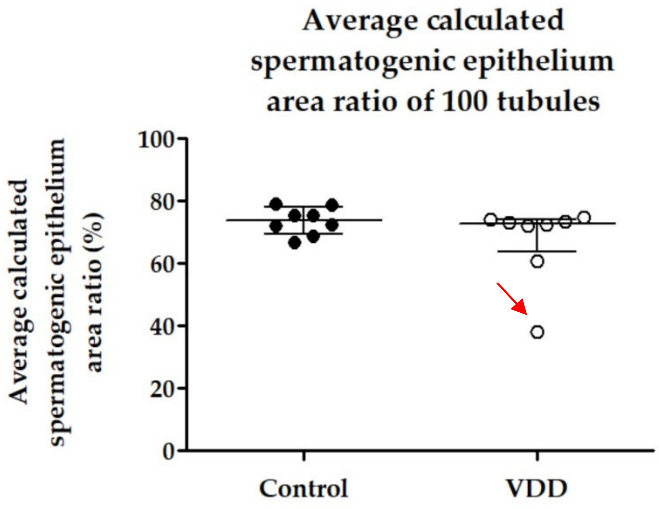

Control/Vitamin D supplemented group (n = 8, %)	Vitamin D deficient/VDD group (n = 8, %)
79.211390	72.594150
66.998090	73.546010
72.623160	74.784300
75.376320	60.954610
72.070430	73.191610
75.449760	38.262200
68.741940	72.310260
78.938330	74.163710

Figure S7 and Table S7: ***Average calculated spermatogenic epithelium area ratio*** per group: Figure S7: in graph, Table S7: original data. Abbreviations: VDD = vitamin D deficient. n = 8/group. Mann-Whitney-U-test, data are in Median [IQR], n.s.

• *Average seminiferous tubule and seminiferous tubule lumen diameter* (µm)

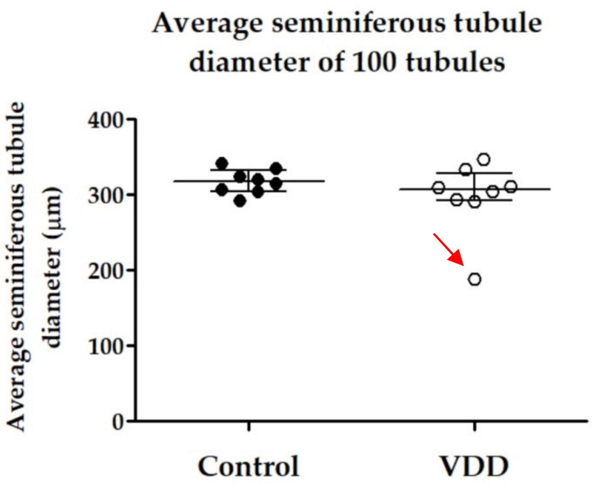

Control/Vitamin D supplemented group (n = 8, µm)	Vitamin D deficient/VDD group (n = 8, µm)
306.8720	334.4965
342.4980	309.8680
304.7790	311.8825
320.7315	294.4070
335.4900	346.9105
314.9790	188.2440
325.2140	291.8850
292.8710	304.2200

Figure S8 and Table S8: *Average seminiferous tubule diameter* per group: Figure S8: in graph, Table S8: original data. Abbreviations: VDD = vitamin D deficient. n =8/group. Mann-Whitney-U-test, data are in Median [IQR], n.s.

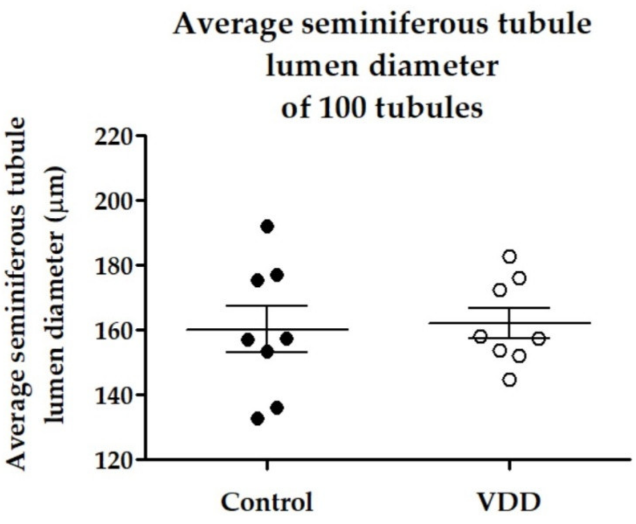

Control/Vitamin D supplemented group (n = 8, µm)	Vitamin D deficient/VDD group (n = 8, µm)
136.0125	172.3830
192.2115	158.1965
157.5620	153.7800
157.1260	182.7540
175.5720	176.2065
153.5840	144.7330
177.0710	152.3010
132.9770	157.3555

Figure S9 and Table S9: *Average seminiferous tubule lumen diameter* per group: Figure S9: in graph, Table S9: original data. Abbreviations: VDD = vitamin D deficient. n = 8/group. Unpaired-t-test, data are in Mean±SEM, n.s.

• *Average spermatogenic epithelium thickness* (μm)

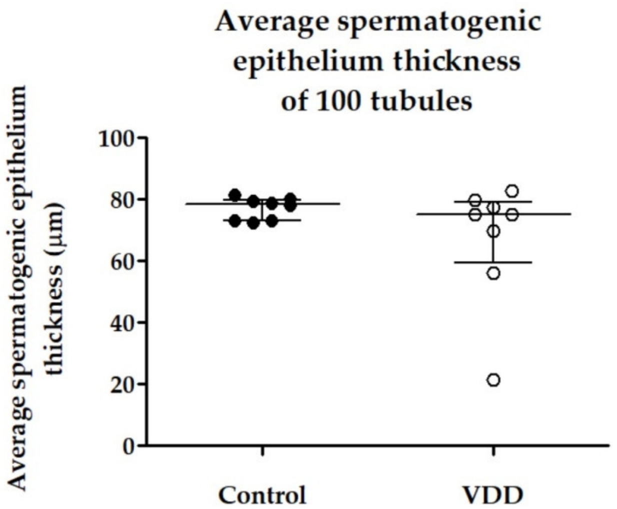

Control/Vitamin D supplemented group (n = 8, μm)	Vitamin D deficient/VDD group (n = 8, μm)
81.532000	79.7232
72.604630	75.1204
73.143600	77.5466
80.129400	56.0182
78.802400	82.9346
79.602800	21.5972
73.252000	69.8434
78.185800	75.2277

Figure S10 and Table S10: *Average spermatogenic epithelium thickness* per group: Figure S10: in graph, Table S10: original data. Abbreviations: VDD = vitamin D deficient. n = 8/group. Mann-Whitney-U-test, data are in Median [IQR], n.s.

According to the figures and tables, there were an extremely outlier in the VDD group in case of seminiferous tubule area, perimeter, diameter and calculated spermatogenic epithelium area, area ratio and thickness (data marked with red arrow in figures and red color in tables). Thus, we would like to show the histological image of that testicular tissue (Picture S1-S2).

As we can see on the pictures, in the 'outlier' tissue, smaller tubule area, perimeter, diameter can be seen, spermatogenic epithelium (SE) is thinner, as well as the SE area and area ratio. The exact measured numbers express the visually-noticeable difference.

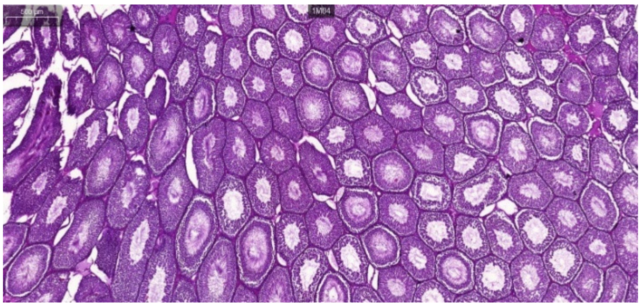

Control

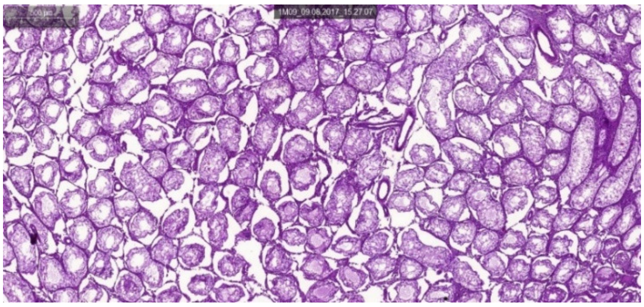

VDD

Picture S1-S2: *Representative histological picture of testicular tissue of one of the control group* (left) *and of the outlier of VDD group* (right). Pictures were made with CaseViewer software. Scale bar 500 μm.

• ***Vitamin D Receptor (VDR)-positively-stained area percentage*** (%)

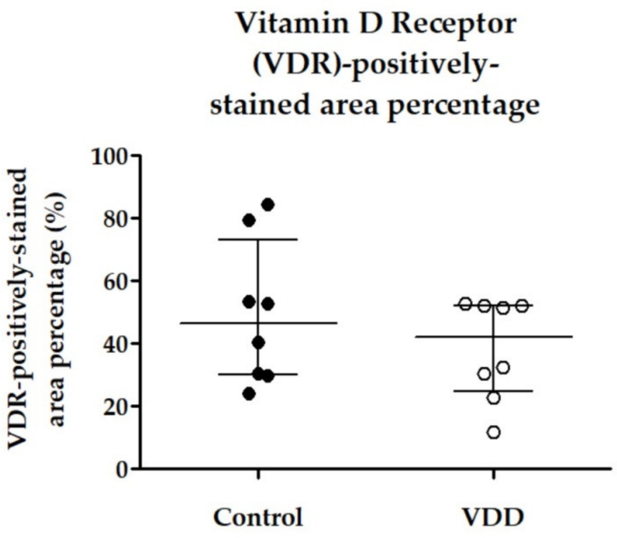

Control/Vitamin D supplemented group (*n* = 8, %)	Vitamin D deficient/VDD group (*n* = 8, %)
30.54311	51.47946
40.40022	11.96844
53.61100	52.04578
52.75968	22.83652
24.15033	52.85780
29.87443	30.60423
79.48771	52.19071
84.57590	32.52902

Figure S11 and Table S11: ***Vitamin D Receptor (VDR)-positively-stained area percentage*** per group: Figure S11: in graph, Table S11: original data. Abbreviations: VDD = vitamin D deficient. *n* = 8/group. Mann-Whitney-U-test, data are in Median [IQR], n.s.

According to our negative results, we hypothesize that short-term (8-week-long) vitamin D deficiency does not affect the structure of the spermatogenic tissue. Further basic, -translational and clinical investigations are required to examine the possible effects of Vitamin D supply and deficiency on testicular tissue, spermatogenesis and the possible role of Vitamin D in male fertility-infertility.

The original manuscript describes a detailed and easy-to-follow methodological description of a suggested, well-reproducible quantitative assessment of testicular tissue.
